# Comparison of functional classification systems

**DOI:** 10.1093/nargab/lqac090

**Published:** 2022-12-01

**Authors:** Monika Zeller, Daniel H Huson

**Affiliations:** Algorithms in Bioinformatics, Institute for Bioinformatics and Medical Informatics, University of Tübingen, Sand 14, 72076 Tübingen, Germany; Algorithms in Bioinformatics, Institute for Bioinformatics and Medical Informatics, University of Tübingen, Sand 14, 72076 Tübingen, Germany

## Abstract

In microbiome analysis, functional profiling is based on assigning reads or contigs to terms or nodes in a functional classification system. There are a number of large, general-purpose functional classifications that are in use, such as eggNOG, KEGG, InterPro and SEED. Smaller, special-purpose classifications include CARD, EC, MetaCyc and VFDB. Here, we compare the different classifications in terms of their overlap, redundancy, structure and assignment rates. We also provide mappings between main concepts in different classifications. For the large classifications, we find that eggNOG performs the best with respect to sequence redundancy and structure, SEED has the cleanest hierarchy, whereas KEGG and InterPro:BP might be more informative for medical applications. We illustrate the practical assignment rates for different classifications using a number of metagenomic samples.

## INTRODUCTION

In microbiome analysis, functional profiling aims at answering the question of ‘what can microorganisms in this sample do?’. One straight-forward way to address this is to align microbiome shotgun sequencing reads (or their assemblies) against functional reference sequences and then to bin them by function (1,2).

There exist a number of large functional classification systems that can be used for general-purpose analysis, such as eggNOG (3), KEGG (4), InterPRO families (5) or SEED (6). Specific questions are sometimes addressed using more special-purpose databases. For example, Enzyme (EC numbers) (7) and MetaCyc (8) can be used to analyze metabolism, whereas CARD (9), MEGARes (10) and ARGminer (11) address antimicrobial resistance, and VFDB (12) focuses on virulence factors.

How do these approaches differ from each other? To compare different functional classification systems, we first looked at their sequence content, that is, the sequences contained in the databases associated with each hierarchy. For each sequence in one database we searched for its best match in another.

Matching can be done by sequence similarity or semantically. Semantic comparison matches proteins by their annotated function, based on assigned GO terms, say, and does not always correlate strongly with sequence similarity. So, potentially, semantic search allows one to find matches that are not detectable by homology search (13). However, since metagenomic data analysis is usually based on homology search methods, such as BLAST (14) or DIAMOND (15); here, we will match sequences based on similarity only. To avoid confusion we did not use any mapping between sequences or terms in databases that are provided by their curators. Instead we performed DIAMOND alignment of sequences present in one database against the other.

We compared four of the most widely used general-purposes databases so as to determine how they cover and complement one another, namely InterPro families (mapped to the GO:Biological Processes part of the metagenomic GO-slim (16), referred to here as InterPro:BP), eggNOG, KEGG pathways and SEED. We explored how well concepts in one system map onto concepts in the other systems. In addition, we compared the assignment rates for the four databases on five different short read datasets sampled from different environments, as calculated using MEGAN6 (17).

Additionally, we investigated how the six mentioned special-purpose classification systems fit into the four presented general-purpose ones.

We found that eggNOG performs the best with respect to sequence redundancy and structure, whereas, KEGG, SEED and InterPro:BP have much higher sequence redundancy. Moreover, the GO:Biological Processes classification, as provided by in the metagenomic GO slim, has a structure that is too general for good overviews.

### General purpose functional classifications

Three of the above mentioned general-purpose classifications (eggNOG, KEGG and SEED) are hierarchically structured whereas some, but not all, of the InterPro families are connected among themselves. In order to get a complete hierarchy, they are connected with GO (18). In each hierarchy root nodes correspond to the database itself, whereas leaves represent the most specific terms and have reference sequences associated with them. This makes it easy to provide overviews at different levels of detail. The most specific terms in eggNOG and KEGG are othologous groups, in InterPro:BP it is protein families and subsystems in SEED. Table 1 summarizes main statistics of the four hierarchies.

**Table 1.** Basic statistics. For each of the classifications eggNOG, KEGG, InterPro:BP and SEED, we report the median depth (that is, the median number of nodes on the path from the root to leaves), the number of internal nodes and leaves, and the number of reference sequences

	eggNOG	KEGG	InterPro:BP	SEED
Median depth	4	5	3	5
Internal nodes	28	591	1528	154
Leaves	30 955	55 124	9581	823
Sequences	7.5M	13.2M	14.8M	47.7M

Protein families include all evolutionary related proteins, namely both othologs and paralogs. While othologs arise due to speciation events and contain proteins that share the same function, paralogs emerge by gene duplication, with a possible subsequent change of function (19). Therefore, orthologous groups are by nature more conserved than protein families. Subsystems, as used by SEED, are even more general than protein families and are more similar to pathways. Each subsystem contains a set of proteins that realize a specific biological process or a structural complex (20). This is reflected in the statistics reported in Table 1. EggNOG and KEGG have fewer sequences, but significantly more leaves than InterPro:BP and SEED, whereas SEED has fewest leaves (subsystems), but most sequences.

All four databases are based on data from various sources as well as on each other. EggNOG which is an acronym for ‘Evolutionary genealogy of genes: Non-supervised Orthologous Groups’ is compiled from genomes that undergo checks for quality and completeness. In addition it contains some manually curated orthologous groups (3). Each orthologous group is assigned a function using a sophisticated pipeline and many different sources including GO (18), KEGG (4) and placed in a functional category as introduced in COG (21), KOG (22) and arCOG (23).

The Kyoto Encyclopedia of Genes and Genomes (KEGG) is a collection of 18 specific databases that are all manually curated, except for one computationally generated database (SSDB) (24). KEGG contains information from fully sequenced genomes as well as individual proteins with experimentally characterized function (4). Here, we considered all KOs from the KEGG othology database except for those of the BRITE hierarchies.

InterPRO families is one of the largest databases for automatic sequence annotation. InterPRO contains 27.5M sequences from 14 other databases (5): CATH-Gene3D (25), CDD (26), HAMAP (27), PANTHER (28), Pfam (29), PIRSF (30), PRINTS (31), ProDom (32), PROSITE Patterns (33), PROSITE Profiles (33), SMART (34), SFLD (35), SUPERFAMILY (36) and TIGRFAMs (37). Data from these databases are manually integrated into InterPRO and a GO number is assigned. We analyzed InterPRO families as mapped to GO slim terms used for metagenomic analyses in MEGAN (17). As explained below in the methods section, of the three ontologies available in GO, we only consider Biological Process.

The SEED contains protein families that are compiled from various resources and contains genes from published genomes only. It uses public genomes annotated by the RAST (Rapid Annotation using Subsystem Technology) pipeline (38), expert user annotations, metabolic modeling data (39,40), expression data and literature references verifying annotations (41). SEED is widely used both for microbial genome annotation in the RAST pipeline and for metagenomic analysis in MG-RAST (2). The hierarchy of functional classification terms behind the SEED is based on subsystems as introduced in (20). The most recent version of it is included in PATRIC (Pathosystems Resource Integration Center) (42) as Subsystems.

### Special-purpose functional classifications

MetaCyc and Enzyme (EC numbers) are two widely used databases (and implicit classifications) that are dedicated to metabolism. MetaCyc is a well curated and evidence-based database of metabolic pathways and enzymes. It contains proteins from experimentally determined and published pathways. Because the curators apply strict criteria, it only contains 12K sequences. In comparison, the Enzyme database contains over 230K proteins. EC numbers are recommended by the Nomenclature Committee of IUBMB (International Union of Biochemistry and Molecular Biology) (43) and the database contains both confirmed and preliminary EC numbers.

Antibiotic resistance is an important medical category. There are a number of databases that address this. Identifying ARGs (antibiotic resistance genes) in a microbiome setting requires a high quality database of reference sequences and a dedicated analysis pipeline (44). The prediction of antibiotic resistance is more challenging than the general-purpose functional profiling discussed above. Antibiotic resistance depends on a number of different features, such as the presence, or absence, of a certain protein variant, or DNA sequence, or SNP, or on the order of genes along the genome, say. CARD (Comprehensive Antibiotic Resistance Database) is the smallest ARG database with 2.6K sequences. It is highly curated and contains only antimicrobial resistance determinants with clear experimental evidence (9). MEGARes contains almost 8K sequences and is also manually curated. It is specifically intended for high-throughput data, which makes it especially applicable to metagenomics studies. The MEGARes database is compiled from non-redundant proteins obtained from CARD (9) and other sources (10). ARGminer contains almost 15K protein sequences (11). It is a community curated resource that is dependent on several other resources, including both CARD (9) and MEGARes (10).

A further topic that is also relevant to medical research is pathogen identification. VFDB (Virulence Factor Database) is a database dedicated to this question. It is well curated and contains data from 32 medically important pathogens. It is available in two versions. The ‘core’ dataset contains only experimentally validated virulence factors, whereas the ‘full’ dataset contains all genes related to known and predicted virulence factors (12).

## MATERIALS AND METHODS

### Data download

Sequence data for functional classification systems was obtained from the following databases: the NCBI non-redundant protein database (NCBI-nr) downloaded January 2021 from ftp://ftp.ncbi.nih.gov/blast/db/FASTA/nr.gz, InterPRO downloaded January 2021 from ftp://ftp.ebi.ac.uk/pub/databases/interpro, eggNOG version 4.5 downloaded January 2021 from http://eggnogdb.embl.de, KEGG orthology downloaded January 2021 from http://rest.kegg.jp, SEED as extracted from PATRIC 3.6.9, Enzyme downloaded July 2020, MetaCyc 24.0, CARD downloaded August 2020, VFDB downloaded August 2020, MegaRES v2.0, ARGMiner v1.1.1.A, ACLAME v0.4. The metagenomic GO slim was downloaded January 2021 from http://geneontology.org/ontology/subsets.

### The three GO domains and InterPro

**Figure 1.**
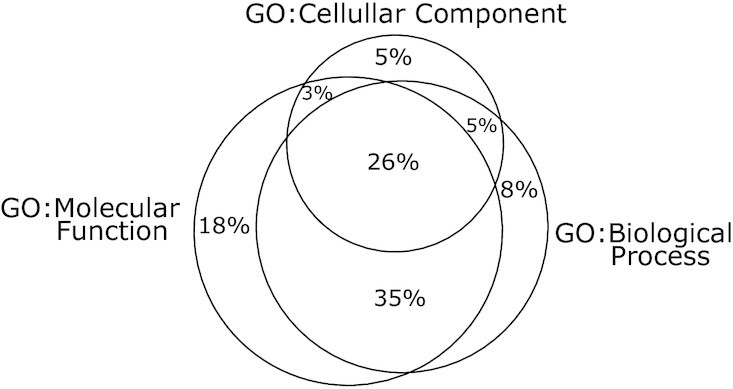
Venn diagram shown the percentage of InterPro family sequences assigned to the three GO domains (biological process, cellular component and molecular function).

The GO gene ontology consists of three separate domains: biological process, cellular component and molecular function (18). All three are also represented in the metagenomic GO-slim used here (16). MEGAN uses a hierarchy based on the metagenomic GO-slim to classify InterPro families. To determine how redundant the representation of InterPro families by the three different domains is, we determined the percentage of proteins in InterPro families that they share, as shown in Figure 1.

The two domains biological process and molecular function contain 95% of all sequences present in InterPro families. Molecular function contains 17M sequences whereas biological process over 15M. Because the GO:Biological Process ontology is conceptually the most similar to other functional classification systems discussed here, we only consider this GO ontology.

Throughout this paper, we use the term InterPro:BP to refer to the functional classification that uses InterPro families as leaves and terms of the metagenomic GO-slim as internal nodes, as introduced in (17), restricted to the GO:Biological Process domain.

### Database redundancy

To determine redundancy, databases discussed in the paper were clustered using CD-HIT (45) version 4.8.1 at a sequence identity threshold of both 0.9 and 0.7, with a length difference cutoff of 0.9 and minimal alignment coverage of 0.9. To verify the obtained number of clusters, USEARCH (46) v11.0.667 was additionally applied to the small databases. On average USEARCH found approximately 0.4% more clusters than CD-HIT (see Supplementary Table S1).

### Comparison of functional classification systems by read mapping

Five examples of short read microbiome shotgun-sequencing datasets were downloaded from the NCBI Sequence Read Archive (SRA) (47), see Table 2. All five datasets were preprocessed with fastp (v0.21.0) (48), with the base quality threshold set to 20 and all other parameters using default values.

**Table 2.** Information on short read data. For each dataset, referred to by its environmental source, we report the Biosample ID, number of reads after quality filtering and average read length

Dataset	Biosample	No. reads	Length
Gut	SAMEA4731558	16.3M	150
Skin	SAMEA4611035	22.8M	100
Water	SAMEA2421979	0.6M	511
Landfill1	SAMN14103086	15.7M	150
Landfill2	SAMN13336098	25.3M	151

To explore the assignment rate of each functional classification system in practical metagenomic analysis, we analyzed the short read datasets using both the general-purpose ‘DIAMOND against NCBI-nr + MEGAN’ pipeline and also a dedicated ‘DIAMOND against a specific database + MEGAN’ pipeline, for each specific database, using DIAMOND (15) (v2.0.7) in BLASTX mode and MEGAN6 (v6.21.1) (17) (default parameters).

To compare read mapping to different functional classification systems we used DIAMOND (query cover set to 90%, otherwise default parameters) retaining only the best match. For each pair of functional classifications, we determined the proportion of reads that could be mapped in both classifications, or only one of them.

### Comparison of functional classification systems by sequence content

To compare functional classification systems as a whole, we mapped all sequences contained in one database on to all others. For consistency, we ignored any mappings provided by the authors of the classification systems, and instead compiled DIAMOND indices for each functional classification system, and then used DIAMOND in BLASTP mode to match sequences. For each sequence only one best hit with at least 90% of query coverage was considered (with default values for all other DIAMOND parameters).

Functional classification systems were compared in pairs. First we determined which proportion of sequences from one database could be mapped on to another. To compare hierarchies of functional classifications, we considered selected nodes or terms, together with all sequences assigned to them and all their descendants. For the different classifications, we decided which hierarchy level to consider based on how general or specific it’s nodes are. We aimed to select nodes that were general enough to provide an overview of the data while remaining functionally specific. Thus, for eggNOG and KEGG we considered the second level below the root, for GO:Biological Process the first level and for SEED a mixture of levels that best matched the level of detail of the other three functional classifications. In eggNOG the choice corresponds to functional categories, in KEGG to smallest groups of pathways, in InterPro:BP to GO terms as available in the metagenomic GO slim whereas in SEED it was a set of superclasses, classes and subclasses as available in the subsystems of PATRIC.

For each pair of nodes from two different classifications, we determined the proportion of sequences assigned to the one node (and its descendants) that had matches to sequences assigned to the other node. The proportion of sequences that could not be assigned to any of the nodes was also determined.

Note that matching between functional classification systems is usually not symmetric because the number of sequences assigned to the compared nodes may be very different, and because sequence alignment is not symmetric.

## RESULTS

### Size and redundancy of functional databases

**Figure 2.**
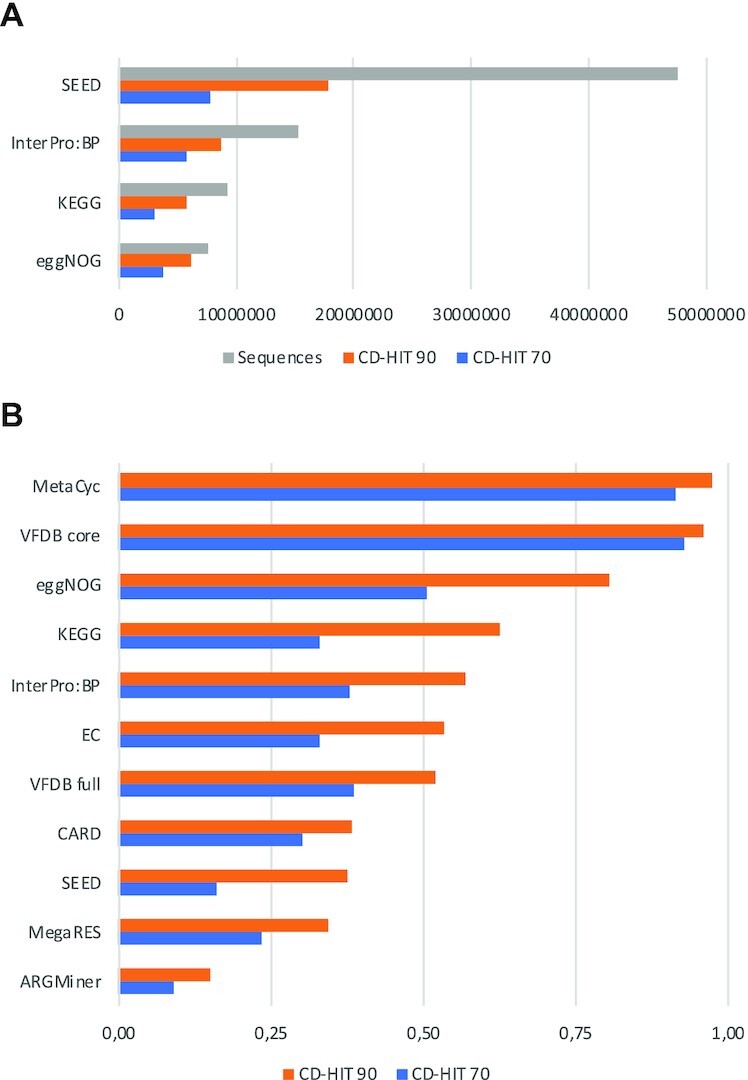
(**A**) For four large functional classification systems, we report the number of reference sequences, and the number of clusters obtained using CD-Hit with a sequence identity cutoff of 90% or 70%, respectively. (**B**) For various functional classification systems, we report the ratio of the number of clusters to reference sequences, for clusters obtained using CD-Hit with a sequence identity cutoff of 90% or 70%, respectively.

To determine the redundancy of each of the four main functional classification systems available in MEGAN (InterPro:BP, eggNOG, KEGG and SEED), we clustered all sequences using a sequence identity threshold of both 90% and 70%. Figure 2 A shows the difference between the number of sequences and the number of clusters. The lower the identity threshold is, the smaller the difference in size among the four large functional classification systems is. For example, SEED has more than six times as many sequences as eggNOG, but only three times as many clusters with 90% and twice as many with 70% sequence identity.

In Figure 2B, we present the clusters-to-sequences ratio in all functional classification systems discussed here (large and small). The number of clusters obtained (for both 90% and 70% identity) indicates that eggNOG has more diversity than KEGG even though it contains less sequences in total. SEED and InterPro:BP both have more clusters than eggNOG; however, SEED has a very low sequence to cluster ratio, indicating a high percentage of sequence similarity and low diversity for the total number of sequences. For exact numbers, please see Supplementary Table S2.

Both MetaCyc and the core of VFDB are small and strictly curated databases, containing only proteins that have been experimentally validated In consequence, they have a high cluster-to-sequence ratio of over 0.96, for 90% identity, and over 0.91, for 70%, respectively. Databases for antimicrobial resistance have the highest redundancy. This is because all variants are of high importance and only hits with very high sequence similarity are of interest.

### Comparison of functional classifications

**Figure 3.**
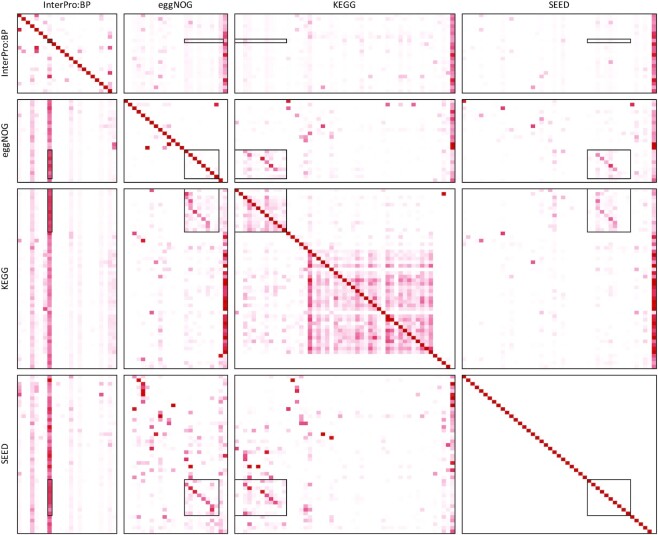
Heatmaps showing how nodes from functional classifications on the left are mapped to the nodes of functional classifications along the top. White indicates 0% sequences, dark red—100%, on a linear scale. The last column in each heatmap shows the proportion of the sequences that cannot be matched to any node. Boxes highlight the nodes that correspond to metabolism (or metabolic process in InterPro:BP). More details can be found in the Supplementary Tables S3–S8.

Table 3 shows the percentage of sequences in each of the four large functional classification systems that can be mapped to each of the others. From this general comparison it appears that InterPro:BP contains most of the information that is present in eggNOG, KEGG pathways and SEED. However, these numbers say nothing about structural similarities among the four. The heatmaps presented in Figure 3 and Supplementary Tables S3–S8 provide a more detailed comparison.

**Table 3.** For each functional classification system listed on the left, we report how well it maps to the classification systems listed along the top, in other words, the percentage of sequences that are covered by sequences in the system listed on the top

	InterPro:BP	eggNOG	KEGG	SEED
InterPro:BP	—	68.1	59.2	55.9
eggNOG	97.7	—	65.3	70.6
KEGG	97.2	75.4	—	64.2
SEED	98.7	97.3	82.7	—

Note that a high percentage of SEED is covered by both eggNOG and InterPro:BP but not the other way around. This is also the case for KEGG, although not as much of it is covered by eggNOG as is by InterPro:BP. This suggests that eggNOG and InterPro:BP are more general than SEED and KEGG, and so we first discuss similarities and differences between eggNOG and InterPro:BP

### Comparison of eggNOG and InterPro:BP

Even though eggNOG maps much better to InterPro:BP, than vice versa, the structure of InterPro:BP appears to be uninformative. Almost all of the nodes map best to GO:0008152 Metabolic Process (approximately 50% sequences per node) and GO:0006810 transport (approximately 20% of sequences per node) (see Supplementary Table S3A). Only two exceptions are present in eggNOG, that is Extracellular Structures and Cytoskeleton. Bot of these nodes have a large proportion (52% and 44%, respectively) of sequences that do not have matches in InterPro:BP. The node GO:0008152 Metabolic Process contains almost 100 other GO terms and InterPro families that are much more specific than eggNOG terms and thus are not suited for a comparison. The structure of InterPro:BP lacks intermediate levels and goes directly from very general to very specific terms, making InterPro:BP less useful than eggNOG for functional overviews.

We observed the same trend for mappings of KEGG and SEED to InterPro:BP (see Supplementary Tables S4 and S5). Therefore we used eggNOG as reference database for exploring KEGG and SEED and will not discuss their similarity to InterPro:BP in detail.

The mapping of InterPro:BP to eggNOG is also very poor (Supplementary Table S3B). Most of the terms are largely unassigned whereas matches that can be found are very weak (less than 30% of sequences), as shown in the Table 4. InterPro:BP terms that have >65% of unassigned sequences are GO:0009405 Pathogenesis (68% unassigned), GO:0016032 Viral Process (74%) and GO:0046718 Viral Entry into Host Cell (73%). This indicates that eggNOG may lack information present in InterPro:BP that is important for medical applications.

**Table 4.** Matching of InterPro:BP terms to eggNOG terms. For each pair, we report the percentage of reference sequences for a InterPro:BP node that are highly similar to some reference sequence for the corresponding eggNOG node. Matches are shown only for those nodes in InterPro:BP that had a best match in eggNOG covering at least 15% of sequences contained in it. The complete list is provided in Supplementary Table S3B

InterPro:BP	eggNOG	%
GO:0006259 DNA metabolic process	Information storage and processing → [L] Replication, recombination and repair	27
GO:0006457 protein folding	Cellular processes and signaling → [O] Post-translational modification, protein turnover, chaperones	27
GO:0006808 regulation of nitrogen utilization	Metabolism → [E] Amino acid transport and metabolism	28
GO:0006950 response to stress	Information storage and processing → [L] Replication, recombination and repair	21
GO:0008152 metabolic process	Metabolism → [C] Energy production and conversion	16
GO:0016070 RNA metabolic process	Information storage and processing → [J] Translation, ribosomal structure and biogenesis	17
GO:0016226 iron-sulfur cluster assembly	Cellular processes and signaling → [O] Post-translational modification, protein turnover, chaperones	23
GO:0019222 regulation of metabolic process	Information storage and processing → [K] Transcription	15
GO:0045454 cell redox homeostasis	Cellular processes and signaling → [O] Post-translational modification, protein turnover, chaperones	30
GO:0071973 bacterial-type flagellum-dependent cell motility	Cellular processes and signaling → [N] Cell motility	33

### Comparison of KEGG and eggNOG

About half of the considered nodes in KEGG have good matches in eggNOG. As shown in Table 5, most of the terms under Metabolism in KEGG map well to Metabolism terms in eggNOG, whereas genetic information processing in KEGG maps to information storage and processing in eggNOG. As can be seen in the heatmap in Figure 3 and in Supplementary Table S6A, KEGG terms are often spread across eggNOG. Most of the nodes under human diseases in KEGG appear to be mostly unassigned in eggNOG indicating that as with InterPro:BP, eggNOG is missing medically relevant information available in KEGG.

**Table 5.** Matching of KEGG terms to eggNOG terms. For each pair, we report the percentage of reference sequences for a KEGG term that are highly similar to some reference sequence for the corresponding eggNOG term. Matches are shown only for those terms in KEGG that had a best match in eggNOG covering at least 15% of sequences contained in it. The complete list is provided in Supplementary Table S6a

KEGG		eggNOG	%
Metabolism	Glycan biosynthesis and metabolism	Cellular processes and signaling → [M] Cell wall/membrane/envelope biogenesis	55
	Not included in regular maps	Metabolism → [C] Energy production and conversion	63
	Energy metabolism	Metabolism → [C] Energy production and conversion	40
	Amino acid metabolism	Metabolism → [E] Amino acid transport and metabolism	56
	Metabolism of other amino acids	Metabolism → [E] Amino acid transport and metabolism	32
	Biosynthesis of other secondary metabolites	Metabolism → [E] Amino acid transport and metabolism	34
	Nucleotide metabolism	Metabolism → [F] Nucleotide transport and metabolism	65
	Carbohydrate metabolism	Metabolism → [G] Carbohydrate transport and metabolism	35
	Metabolism of cofactors and vitamins	Metabolism → [H] Coenzyme transport and metabolism	45
	Lipid metabolism	Metabolism → [I] Lipid transport and metabolism	41
	Metabolism of terpenoids and polyketides	Metabolism → [I] Lipid transport and metabolism	38
	Xenobiotics biodegradation and metabolism	Metabolism → [I] Lipid transport and metabolism	19
Genetic Information Processing	Translation	Information storage and processing → [J] Translation, ribosomal structure and biogenesis	58
	Transcription	Information storage and processing → [K] Transcription	16
	Replication and repair	Information storage and processing → [L] Replication, recombination and repair	75
	Folding, sorting and degradation	Cellular processes and signaling → [U] Intracellular trafficking, secretion and vesicular transport	17
Environmental information processing	Membrane transport	Metabolism → [P] Inorganic ion transport and metabolism	25
	Signal transduction	Cellular processes and signaling → [T] Signal transduction mechanisms	18
Cellular processes	Cell motility	Cellular processes and signaling → [N] Cell motility	43
	Cellular community - prokaryotes	Metabolism → [E] Amino acid transport and metabolism	27
Organismal systems	Aging	Cellular processes and signaling → [O] Post-translational modification, protein turnover, chaperones	38
Human diseases	Drug resistance: antimicrobial	Cellular processes and signaling → [M] Cell wall/membrane/envelope biogenesis	52
	Cardiovascular disease	Cellular processes and signaling → [O] Post-translational modification, protein turnover, chaperones	28
	Drug resistance: antineoplastic	Cellular processes and signaling → [O] Post-translational modification, protein turnover, chaperones	16
Not included in pathway or brite	Poorly characterized	Poorly characterized → [S] Function unknown	83
	Unclassified: genetic information processing	Information storage and processing → [L] Replication, recombination and repair	47
	Unclassified: metabolism	Poorly characterized → [S] Function unknown	21
	Unclassified: signaling and cellular processes	Metabolism →[P] Inorganic ion transport and metabolism	17

The reverse mapping of eggNOG to KEGG (Table 6 and Supplementary Table S6B) shows a similar trend with respect to metabolism and information storage and processing nodes. Except for [B] Chromatin structure and dynamics, hardly any nodes in eggNOG match to Human Diseases in KEGG. Even though KEGG has more sequences than eggNOG, a larger part of eggNOG is unmapped to KEGG than in the opposite direction. This confirms that eggNOG has a greater variety of sequences and is less redundant than KEGG.

**Table 6.** Matching of eggNOG terms to KEGG terms. For each pair, we report the percentage of reference sequences for the first term that are highly similar to some reference sequence for the second term. Matches are shown only for those terms in eggNOG that had a best match in KEGG covering at least 15% of sequences contained in it. The complete list is provided in Supplementary Table S6B

eggNOG		KEGG	%
Information Storage And Processing	[A] RNA processing and modification	Genetic Information Processing → Translation	76
	[B] Chromatin structure and dynamics	Human Diseases → Cancer: overview	50
	[J] Translation, ribosomal structure and biogenesis	Genetic Information Processing → Translation	50
	[L] Replication, recombination and repair	Genetic Information Processing → Replication and repair	34
Cellular Processes And Signaling	[M] Cell wall/membrane/envelope biogenesis	Metabolism → Glycan biosynthesis and metabolism	24
	[N] Cell motility	Cellular Processes → Cell motility	59
	[T] Signal transduction mechanisms	Environmental Information Processing → Signal transduction	47
	[U] Intracellular trafficking, secretion, and vesicular transport	Environmental Information Processing → Membrane transport	36
	[V] Defense mechanisms	Environmental Information Processing → Membrane transport	44
Metabolism	[C] Energy production and conversion	Metabolism → Energy metabolism	34
	[E] Amino acid transport and metabolism	Metabolism → Amino acid metabolism	47
	[F] Nucleotide transport and metabolism	Metabolism → Nucleotide metabolism	75
	[G] Carbohydrate transport and metabolism	Metabolism → Carbohydrate metabolism	43
	[H] Coenzyme transport and metabolism	Metabolism → Metabolism of cofactors and vitamins	69
	[I] Lipid transport and metabolism	Metabolism → Lipid metabolism	44
	[P] Inorganic ion transport and metabolism	Environmental Information Processing → Membrane transport	28
Poorly Characterized	[S] Function unknown	Not Included in Pathway or Brite → Poorly characterized	16

### Comparison of SEED and eggNOG

Out of all mappings that we considered here, SEED to eggNOG has the best and most meaningful matches. See Table 7 for the best matches from SEED to eggNOG (and Supplementary Table S7A for all matches). SEED appears to have a more user-friendly structure with terms that are slightly more specific than in eggNOG, but that are still general enough to provide a good overview of the data.

**Table 7.** Matching of SEED terms to eggNOG terms. For each pair, we report the percentage of reference sequences for the first term that are highly similar to some reference sequence for the second terms. Matches are shown only for those terms in SEED that had a best match in eggNOG covering at least 15% of sequences contained in it. The complete list is provided in Supplementary Table S7A

SEED		eggNOG	%
RNA processing	RNA processing and modification	Information storage and processing → [J] Translation, ribosomal structure and biogenesis	39
	Transcription	Information storage and processing → [K] Transcription	87
Experimental subsystems		Information storage and processing → [L] Replication, recombination and repair	75
DNA processing	DNA recombination	Information storage and processing → [L] Replication, recombination and repair	58
	DNA repair	Information storage and processing → [L] Replication, recombination and repair	84
	DNA replication	Information storage and processing → [L] Replication, recombination and repair	90
	DNA sulfur modification	Metabolism → [P] Inorganic ion transport and metabolism	21
	DNA uptake, competence	Information storage and processing → Replication, recombination and repair	52
	Type I restriction-modification systems	Cellular processes and signaling → [V] Defense mechanisms	95
Protein processing	Protein Synthesis	Information storage and processing → [J] Translation, ribosomal structure and biogenesis	81
	Protein degradation	Cellular processes and signaling → [O] Posttranslational modification, protein turnover, chaperones	42
	Protein folding	Cellular processes and signaling → [O] Posttranslational modification, protein turnover, chaperones	94
	Protein processing and modification	Cellular processes and signaling → [O] Posttranslational modification, protein turnover, chaperones	34
	Protein targeting, sorting, translocation	Cellular processes and signaling → [U] Intracellular trafficking, secretion, and vesicular transport	55
	Protein glycosylation in prokaryotes	Cellular processes and signaling → [M] Cell wall/membrane/envelope biogenesis	52
	Selenoproteins	Metabolism → [E] Amino acid transport and metabolism	32
Cellular processes	Motility and chemotaxis	Cellular processes and signaling → [N] Cell motility	74
	Microbial communities	Metabolism → [G] Carbohydrate transport and metabolism	32
	Cell cycle, cell division and death	Cellular processes and signaling → [M] Cell wall/membrane/envelope biogenesis	27
Cell envelope		Cellular processes and signaling → [M] Cell wall/membrane/envelope biogenesis	69
Energy	Energy and precursor metabolites generation	Metabolism → [C] Energy production and conversion	44
	Respiration	Metabolism → [C] Energy production and conversion	64
Clustering-based subsystems	Cell division	Information storage and processing → [K] Transcription	23
	Lysine biosynthesis	Metabolism → [E] Amino acid transport and metabolism	86
	Possible heterocyst differentiation related cluster	Cellular processes and signaling → [M] Cell wall/membrane/envelope biogenesis	30
	Shikimate kinase SK3 cluster	Poorly characterized → [S] Function unknown	40
	Threonine synthase cluster	Metabolism → [E] Amino acid transport and metabolism	52
Metabolism	Metabolite damage and its repair or mitigation	Metabolism → [F] Nucleotide transport and metabolism	27
	Nitrogen metabolism	Metabolism → [C] Energy production and conversion	48
	Amino acids and derivatives	Metabolism → [E] Amino acid transport and metabolism	73
	Nucleosides and nucleotides	Metabolism → [F] Nucleotide transport and metabolism	73
	Carbohydrates	Metabolism → [G] Carbohydrate transport and metabolism	42
	Cofactors, vitamins, prosthetic groups	Metabolism → [H] Coenzyme transport and metabolism	50
	Fatty acids, lipids and isoprenoids	Metabolism → [I] Lipid transport and metabolism	53
	Iron acquisition and metabolism	Metabolism → [P] Inorganic ion transport and metabolism	52
	Phosphate Metabolism	Metabolism → [P] Inorganic ion transport and metabolism	68
	Sulfur Metabolism	Metabolism → [P] Inorganic ion transport and metabolism	27
Regulation and cell signaling		Cellular Processes And Signaling → [T] Signal transduction mechanisms	49
Membrane transport		Metabolism → [P] Inorganic ion transport and metabolism	29
Secondary metabolism		Metabolism → [Q] Secondary metabolites biosynthesis, transport and catabolism	23
Miscellaneous		Poorly Characterized → [S] Function unknown	36

The reverse mapping as shown in the Table 8 is not as good (see also Supplementary Table S7B). With only five nodes having best matches covering at least 50% of sequences. This is in line with the large redundancy in SEED reported in Figure 2.

**Table 8.** Matching of eggNOG terms to SEED terms. For each pair, we report the percentage of reference sequences for the first term that are highly similar to some reference sequence for the second term. Matches are shown only for those terms in eggNOG that had a best match in SEED covering at least 15% of sequences contained in it. The complete list is provided in Supplementary Table S7B

eggNOG		SEED	%
Information storage and processing	[A] RNA processing and modification	RNA processing → RNA processing and modification	75
	[B] Chromatin structure and dynamics	Regulation and cell signaling	69
	[J] Translation, ribosomal structure and biogenesis	Protein processing → Protein synthesis	62
	[L] Replication, recombination and repair	DNA processing → DNA repair	29
Cellular processes and signaling	[D] Cell cycle control, cell division, chromosome partitioning	Cellular processes → Cell cycle, cell division and death	34
	[M] Cell wall/membrane/envelope biogenesis	Cell envelope	15
	[N] Cell motility	Motility and chemotaxis	60
	[O] Post-translational modification, protein turnover, chaperones	Protein processing → Protein folding	16
	[U] Intracellular trafficking, secretion and vesicular transport	Membrane transport	29
	[V] Defense mechanisms	Membrane transport	15
Metabolism	[C] Energy production and conversion	Energy → Respiration	23
	[E] Amino acid transport and metabolism	Metabolism → Amino acids and derivatives	40
	[F] Nucleotide transport and metabolism	Metabolism → Nucleosides and nucleotides	43
	[G] Carbohydrate transport and metabolism	Metabolism → Carbohydrates	26
	[H] Coenzyme transport and metabolism	Metabolism → Cofactors, vitamins, prosthetic groups	65
	[I] Lipid transport and metabolism	Metabolism → Fatty acids, lipids, and isoprenoids	35
	[P] Inorganic ion transport and metabolism	Membrane Transport	24
	[Q] Secondary metabolites biosynthesis, transport and catabolism	Metabolism → Cofactors, vitamins, prosthetic groups	18

### Comparison of SEED and KEGG

As expected, we obtained a much better mapping of SEED to KEGG than vice versa. As shown in Figure 3 and Supplementary Table S8B, a large proportion of the nodes in KEGG can not be mapped at all. Nodes with matches are listed in Table 10. There is a correspondence between metabolism nodes in both classification systems, however other terms appear to be quite scattered. Matches of SEED nodes to Organismal Systems and Human Diseases in KEGG are very weak, indicating that KEGG is better suited than SEED for analyses in a medical context, see Table 9.

**Table 9.** Matching of SEED terms to KEGG terms. For each pair, we report the percentage of reference sequences for the first term that are highly similar to some reference sequence for the second term. Matches are shown only for those terms in SEED that had a best match in KEGG covering at least 15% of sequences contained in it. The complete list is provided in Supplementary Table S8A

SEED		KEGG	%
RNA Processing	RNA processing and modification	Genetic information processing → Folding, sorting and degradation	24
	Transcription	Genetic information processing → Transcription	93
DNA Processing	DNA recombination	Genetic information processing → Replication and repair	40
	DNA repair	Genetic information processing → Replication and repair	68
	DNA replication	Human diseases → Drug resistance: antineoplastic	22
	DNA uptake, competence	Genetic information processing → Replication and repair	44
Protein Processing	Protein synthesis	Genetic information processing → Translation	64
	Protein degradation	Cellular processes → Cell growth and death	23
	Protein folding	Genetic information processing → Folding, sorting and degradation	31
	Protein processing and modification	Not included in pathway or brite → Unclassified: metabolism	35
	Protein targeting, sorting, translocation	Genetic information processing → Folding, sorting and degradation	56
	Protein glycosylation in Prokaryotes	Not included in pathway or Brite → Unclassified: metabolism	41
	Selenoproteins	Metabolism → Metabolism of other amino acids	34
Cellular Processes	Motility and Chemotaxis	Cellular Processes → Cell motility	92
	Microbial communities	Cellular Processes → Cellular community - prokaryotes	84
	Cell cycle, cell division and death	Metabolism → Glycan biosynthesis and metabolism	21
	Prokaryotic cell type differentiation	Not Included in Pathway or Brite → Unclassified: signaling and cellular processes	58
Cell Envelope		Metabolism → Glycan biosynthesis and metabolism	59
Energy	Energy and precursor metabolites generation	Metabolism → Carbohydrate metabolism	78
	Photosynthesis	Metabolism → Energy metabolism	57
	Respiration	Metabolism → Energy metabolism	58
Clustering-based subsystems	Cell division	Metabolism → Metabolism of cofactors and vitamins	15
	Lysine biosynthesis	Metabolism → Amino acid metabolism	85
	Possible heterocyst differentiation related cluster	Not Included in Pathway or Brite → Unclassified: signaling and cellular processes	30
	Shikimate kinase SK3 cluster	Not Included in Pathway or Brite → Poorly characterized	35
	Threonine synthase cluster	Metabolism → Amino acid metabolism	56
Metabolism	Metabolite damage and its repair or mitigation	Metabolism → Nucleotide metabolism	35
	Nitrogen metabolism	Metabolism → Energy metabolism	55
	Amino acids and derivatives	Metabolism → Amino acid metabolism	76
	Nucleosides and nucleotides	Metabolism → Nucleotide metabolism	78
	Carbohydrates	Metabolism → Carbohydrate metabolism	55
	Cofactors, vitamins, prosthetic groups	Metabolism → Metabolism of cofactors and vitamins	62
	Fatty acids, lipids and isoprenoids	Metabolism → Lipid metabolism	57
	Iron acquisition and metabolism	Environmental information processing → Membrane transport	33
	Phosphate metabolism	Environmental Information Processing → Membrane transport	52
	Sulfur metabolism	Metabolism → Energy metabolism	54
Regulation and cell signaling		Metabolism → Nucleotide metabolism	18
Membrane transport		Environmental information processing → Membrane transport	25
Secondary metabolism		Metabolism → Amino acid metabolism	46
Miscellaneous		Not included in pathway or brite → Unclassified: metabolism	18

**Table 10.** Matching of KEGG terms to SEED terms. For each pair, we report the percentage of reference sequences for a KEGG node that are highly similar to some reference sequence for the corresponding eggNOG node. Matches are shown only for those nodes in KEGG that had a best match in SEED covering at least 15% of sequences contained in it. The complete list is provided in Supplementary Table S6A

KEGG		SEED	%
Metabolism	Glycan biosynthesis and metabolism	Cell envelope	26
	Not included in regular maps	Metabolism → Amino acids and derivatives	28
	Energy metabolism	Energy → Respiration	24
	Amino acid metabolism	Metabolism → Amino acids and derivatives	47
	Metabolism of other amino acids	Metabolism → Amino acids and derivatives	17
	Biosynthesis of other secondary metabolites	Metabolism → Amino acids and derivatives	25
	Nucleotide metabolism	Metabolism → Nucleosides and nucleotides	40
	Carbohydrate metabolism	Energy → Energy and precursor metabolites generation	32
	Metabolism of cofactors and vitamins	Metabolism → Cofactors, vitamins, prosthetic groups	52
	Lipid metabolism	Metabolism → Fatty acids, lipids and isoprenoids	35
	Metabolism of terpenoids and polyketides	Metabolism → Fatty acids, lipids and isoprenoids	28
Genetic Information Processing	Translation	Protein processing → Protein synthesis	62
	Transcription	RNA processing → Transcription	22
	Replication and repair	DNA processing → DNA repair	47
Environmental Information Processing	Membrane transport	Membrane transport	21
Cellular Processes	Cell motility	Cellular processes → Motility and chemotaxis	52
	Cellular community - prokaryotes	Membrane transport	28
Organismal Systems	Aging	Stress response, defense and virulence	25
Human Diseases	Drug resistance: antimicrobial	Membrane transport	32
	Cardiovascular disease	Stress response, defense and virulence	15
	Drug resistance: antineoplastic	Stress response, defense and virulence	16

### Self mappings

How well does each functional classification system map to itself? To investigate this, we determined how many sequences are shared among nodes of the same functional classification system. Apart from the expected perfect matches along the diagonals, as shown in Figure 3 and Supplementary Tables S13A–D, we also find some overlaps among different nodes. In InterPro:BP this is explained by the fact that the classification is constructed from a directed acyclic graph and is not based on a hierarchy. This leads to some nodes appearing multiple times on the tree and thus duplicated occurrences of the same terms and sequences.

The eggNOG classification has very few pairs of terms or nodes that ‘intersect’, that is, share reference sequences. For example, [W] Extracellular structures are completely contained in [U] Intracellular trafficking, secretion and vesicular transport. In fact [W] Extracellular structures contain only one COG (COG5295 domain protein) that is also contained in the [U].

While KEGG is hierarchically structured, the same KEGG orthology groups may appear in different metabolic pathways, giving rise to sequences that are associated with multiple nodes. For the two groups ‘Metabolism’ and ‘Environmental Information Processing, Cellular Processes, Organismal Systems and Human Diseases’, that are a lot of intersections between nodes in the same group.

For SEED, all intersections among nodes are empty, indicating a strictly hierarchical classification.

### Illustration of classifications on short read data

When it comes to functional classification of reads there are two questions of interest. The first question is: how do functional classification systems differ with respect to the number of reads that can be mapped to them? In other words, to what extend do functional classification systems overlap and complement each other when it comes to actual data analysis. To explore this, we considered all four large databases and counted reads with significant matches.

**Figure 4.**
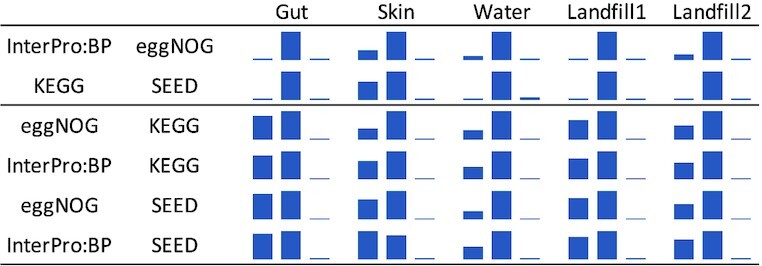
For each pair of functional classifications *A* and *B* listed on the left, and for five different metagenomic sequencing samples (labeled Gut, Skin, Water, Landfill 1 and Landfill 2), we display a bar chart indicating the proportion of reads assigned only in classification *A* (left), in both *A* and *B* (middle), and only in *B* (right), respectively.

The results are reported in Figure 4. There are two patterns. In the first two listed pairs of functional classifications, both classifications roughly covered each other. InterPro:BP shares most of the assigned reads with eggNOG and KEGG with SEED. In the other cases, the first classification complements the other but not vice versa. This is true for all pairs that involve KEGG or SEED but not both. Here about half of the reads that can be mapped to either of the two, have matches in KEGG or SEED whereas almost all mapped reads have matches in either InterPro:BP or eggNOG.

The second question is how sensitive is a functional analysis, as performed by MEGAN, say, on the output of a DIAMOND alignment of a metagenomic sample against the whole NCBI-nr database, in comparison to such an analysis performed on alignments against a special-purpose database that only contains reference sequences for a specific functional classification.

**Figure 5.**
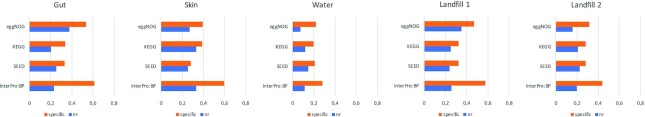
Percentage of reads mapped to functional classification databases with the basic DIAMOND+MEGAN pipeline. Alignment was performed either against a classification specific database (labeled specific) or the whole of NCBI-nr (labeled nr).

To address this, we analyzed all five metagenome samples, using all four main functional classifications, twice each, once based on an alignment of all reads against the NCBI-nr database, and once based on an alignment of all reads against a database containing all references sequences for the functional classification. Functional analysis was performed using MEGAN (default parameters). The 5 × 4 × 2 results are plotted in Figure 5. In all cases, alignment against a specific database gave rise to substantially more mapped reads than alignment against NCBI-nr. Also, more reads could be assigned to InterPro:BP than to the other other three classifications, and more to eggNOG than to KEGG or SEED.

Based on these comparisons, the databases can be ranked as follows: InterPro:BP > eggNOG > KEGG > SEED.

### Taxonomic composition of functional databases

**Figure 6.**
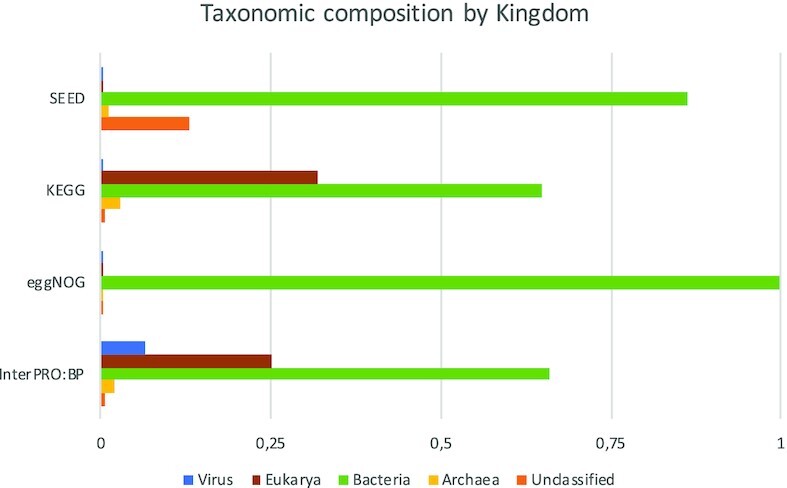
Sequence distribution with respect to taxonomy in the four large functional classification systems.

The above analysis suggests that mappings of SEED and KEGG to eggNOG have much lower assignment rates that to InterPro:BP. However, in practical applications on metagenomic data eggNOG appears to perform almost as well as InterPro:BP. To explore this, we looked into taxonomic composition of all four functional classification systems. Figure 6 shows how sequences within each database are distributed among archeae, bacteria, eukarya and viruses. A large proportion of sequences in all databases are bacterial, however eggNOG is almost exclusively bacterial (99.7% of all sequences). SEED contains sequences that belong to unclassified organisms that are probably bacteria, whereas KEGG and InterPro:BP include sequences from eukaryotic organisms and viruses.

Next we looked at which proportion of bacterial sequences in InterPro:BP, KEGG and SEED could have been mapped to eggNOG and compared it to the proportion of total sequences that have matches in it. As shown in the Table 3, 68.1% of all sequences in InterPro:BP, 75.4% in KEGG and 97.3% in SEED have matches in eggNOG. Table 11 shows which proportion of sequences that belong to different kingdoms have matches in eggNOG. Whereas the number of matched bacterial sequences in SEED is almost the same as for all sequences, it is much higher in KEGG and InterPro:BP. Hence, eggNOG is just as well suited for microbiome analyses as larger functional classification systems are.

**Table 11.** Percentage of sequences originating from different Kingdoms in InterPro:BP, KEGG and SEED that have matches in eggNOG

	InterPro:BP	KEGG	SEED
Unclassified	63.4	73.2	97.4
Archaea	67.5	76.2	77.2
Bacteria	91.4	98.2	97.5
Eukarya	41.9	63.8	93.6
Virus	4.5	80.0	82.8

**Figure 7.**
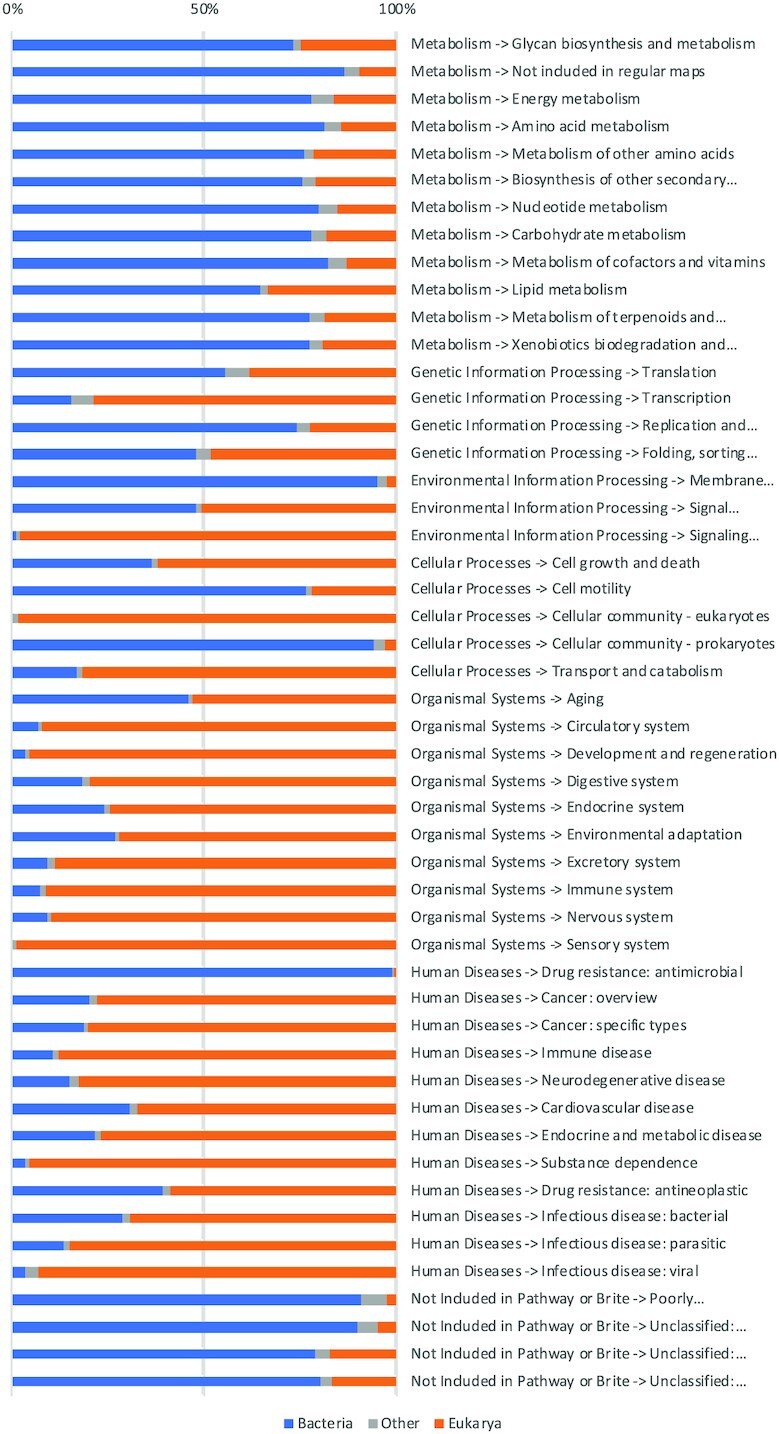
Proportions of bacterial, eukaryotic and other sources of sequences for nodes in KEGG that were used in comparisons against other functional classification systems.

**Figure 8.**
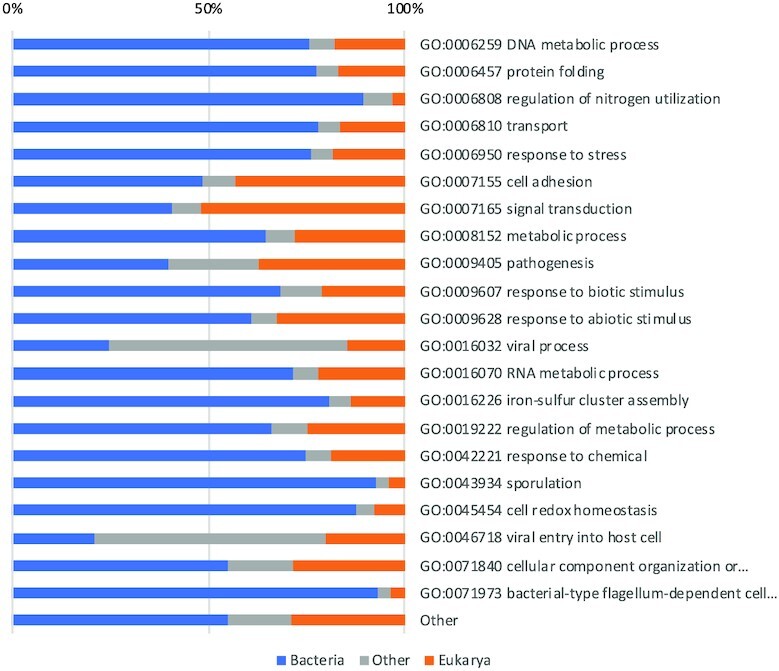
Proportions of bacterial, eukaryotic and other sources of sequences for nodes in InterPro:BP that were used in comparisons against other functional classification systems.

To explore whether medically relevant nodes in KEGG and InterPro:BP are of interest for metagenomic studies, we looked at the taxonomic composition of all nodes in the two as considered in our comparisons. Figure 7 shows taxonomic composition for KEGG. Except ‘Drug resistance: antimicrobial’, all other medically relevant terms under organismal systems and human diseases have more sequences from Eukaryotic organisms than from Bacteria, however the amount of bacterial sequences makes those two categories just as relevant for metagenomics research as other KEGG terms. Regarding medically relevant nodes in InterPro:BP, none of them is dominated by eukaryotic sequences as shown in the Figure 8.

### Small specific databases

All three antibiotic resistance databases map well to eggNOG → [V] Defense mechanisms, KEGG → Drug resistance: antimicrobial, KEGG → Protein families: signaling and cellular processes and SEED → Stress Response, Defense, Virulence. In InterPro:BP the mappings are not so specific. Most of the sequences can be assigned to GO:0008152 metabolic process, GO:0006810 transport and GO:0042221 response to chemical.

The metabolic databases Enzyme and MetaCyc are spread across nodes under eggNOG → Metabolism, KEGG → Metabolism, SEED → Metabolism and GO:0008152 metabolic process.

VFDB maps best to nodes associated with cell membranes, cell motility and intracellular transport in all large functional classification systems.

Out of the seven small databases considered here, VFDB core and VFDB full have the largest proportion of unmapped sequences in eggNOG, KEGG and SEED. InterPro:BP contains matching sequences for almost all of the small databases with only 1–4% of unmatched sequences per database. SEED on the other hand performs worst with 19% to 54% of missing matches. See Supplementary Tables S9–S12 for more details.

## DISCUSSION

In this paper we considered a variety of aspects for analysing and comparing functional classification systems relevant for metagenomic analyses. The size of the database proved not to be a reliable indicator for its functionality. Clustering at 90% and 70% sequence identity shows that the three largest databases (InterPro:BP, SEED and KEGG) are much more redundant than eggNOG, which is smallest of the four. This explains why we were able to map more reads to eggNOG than to SEED or KEGG, even though both are larger than eggNOG.

We noticed that the four large functional classification systems differ much more in their hierarchical structure than in their sequence content. The term Metabolism appears in eggNOG, KEGG and SEED and can be mapped well among the three. However, the term Metabolic Process in InterPro:BP is much more general and does not map as well. In general we found that terms in InterPro:BP are either too general or too specific and thus not well suited for providing an overview. In contrast, eggNOG provides a hierarchy with evenly distributed sequences and provides the best structure for the amount of sequence content. KEGG and SEED are also well structured, however they appear to contain less information with fewer read assignments than eggNOG or InterPro:BP.

Even though we would recommend using eggNOG for general data overviews, it might be less useful when it comes to medical applications. Medically relevant KEGG terms are spread across nodes in eggNOG whereas such terms in InterPro:BP remain largely unassigned. Therefore KEGG and InterPro:BP might be more informative for medical applications.

There are other aspects that we have not considered here that might play a role when choosing a functional classification system. For example, the visualizations of metabolic pathways provided by KEGG may help to analyze the metabolic potential of a sample.

Small specific databases are mostly contained within the four larger ones and can be mapped to the nodes with names that reflect their function. This, however, does not mean that general databases could replace specialized ones. For one, they posses different hierarchical structures that may be more suitable for answering various questions. Furthermore, as shown in the examples in the Figure 5, due to the limited number of hits reported by a homology search, it is more likely that more reads get meaningful assignments when a small specific database is chosen rather than a large one. On the other hand, using a small database increases chances of false positive hits, which might lead to inaccurate interpretations.

## DATA AVAILABILITY

All scripts used in this study are available on Code Ocean (DOI 10.24433/CO.3467358.v1) (49). Short read data can be downloaded from Sequence Read Archive: https://www.ncbi.nlm.nih.gov/sra. MEGAN and the MEGAN mapping database (megan-map-Jan2021.db) are available from https://software-ab.informatik.uni-tuebingen.de/download/megan6. All sequence files for small databases are available from their respective websites.

## Supplementary Material

lqac090_Supplemental_File
